# Quantitative flow ratio (QFR) identifies functional relevance of non-culprit lesions in coronary angiographies of patients with acute myocardial infarction

**DOI:** 10.1007/s00392-021-01897-w

**Published:** 2021-07-12

**Authors:** Andrea Milzi, Rosalia Dettori, Nikolaus Marx, Sebastian Reith, Mathias Burgmaier

**Affiliations:** 1 Department of Cardiology, University Hospital, RWTH Aachen University, Aachen, Germany; 2 Department of Internal Medicine I, University Hospital of the RWTH Aachen, Pauwelsstr. 30, 52074 Aachen, Germany

**Keywords:** Quantitative flow ratio, Coronary artery disease, Acute coronary syndrome, Non-culprit lesion, Coronary physiology

## Abstract

**Introduction:**

In patients with acute myocardial infarction (AMI) and multivessel coronary disease, revascularization of non-culprit lesions guided by proof of ischemia usually requires staged ischemia testing. Quantitative flow ratio (QFR) has been shown to be effective in assessing the hemodynamic relevance of lesions in stable coronary disease. However, its suitability in AMI patients is unknown. In this study, we tested the diagnostic value of QFR based on acute angiograms (aQFR) during AMI to assess the hemodynamic relevance of non-culprit lesions.

**Methods:**

We retrospectively assessed the diagnostic efficiency of aQFR in 280 vessels from 220 patients, comparing it with staged ischemia testing using elective coronary angiography with FFR (*n* = 47), stress cardiac MRI (*n* = 200) or SPECT (*n* = 33).

**Results:**

aQFR showed a very good diagnostic efficiency (AUC = 0.887, 95% CI 0.832–0.943, *p* < 0.001) in predicting ischemia of non-culprit lesions, significantly superior to coronary lesion’s geometry as assessed by quantitative coronary angiography. The optimal cut-off for aQFR to predict ischemia was 0.80 (sensitivity = 83.7%, specificity = 86.1%). Maintaining a predefined level of 95% sensitivity and specificity, we created a decision model based on aQFR: lesions with aQFR ≤ 0.75 should be treated, lesions with aQFR ≥ 0.92 do not yield any hemodynamic relevance, and lesions in the “grey zone” (aQFR 0.75–0.92) benefit from further ischemia testings. This model would allow to reduce staged ischemia tests by 46.8% without a relevant loss in diagnostic efficiency.

**Conclusion:**

Our data demonstrate that aQFR allows an effective assessment of hemodynamic relevance of non-culprit lesions in AMI and may guide interventions of non-culprit coronary lesions.

**Graphic abstract:**

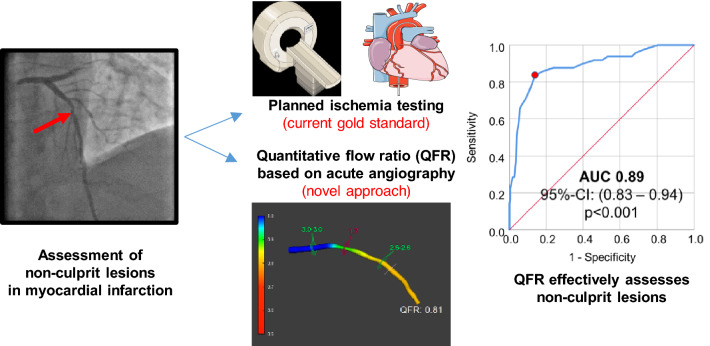

## Introduction

Acute myocardial infarction (MI) represents the acute presentation of coronary artery disease (CAD), and is defined as evidence of myocardial injury with abnormal biomarkers in the setting of acute myocardial ischemia [1]. Invasive coronary angiography plays a central role in modern management of MI, which can confirm obstructive CAD, identify culprit lesion(s) and treat these lesion using percutaneous coronary intervention (PCI).

Previous studies demonstrated that 40–80% of patients presenting with MI have multivessel CAD [2–4]. In patients with multivessel CAD and in the presence of several relevant lesions, complete revascularization has been shown to improve prognosis [2–9]. The evidence regarding the prognostic benefit of a complete revascularization is more robust for patients presenting with STEMI, being based on several randomized studies [2–5, 7–9]; though, some large observational studies suggested a similar effect also in patients presenting with NSTEMI [6, 10–12]. However, in intermediate non-culprit coronary stenoses, the assessment of functional relevance is not possible with the sole use of coronary angiography and may require future ischemia testing including fractional flow reserve (FFR) or non-invasive testing such as stress cardiac magnetic resonance imaging (MRI) or single-photon emission computed tomography (SPECT). This implies further invasive or non-invasive testing, which may be associated to prolonged/another hospitalization, increased costs and/or possible complications (albeit the latter very rarely). Furthermore, staged testing needs the interplay of various actors (among others: the hospital performing the acute angiography, outpatient cardiologists and general practitioners) to be performed and interpreted correctly; this may occasionally lead to suboptimal delivery of care to the patient. Therefore, an interesting option to assess hemodynamic relevance of intermediate coronary lesions may be quantitative flow ratio (QFR), a novel technique able to assess the pressure drop in the vessel based on two angiographic projections. QFR has already been tested in stable CAD as a reliable, safe and cost-effective alternative to FFR to assess functional relevance of coronary stenosis [13–16]; however, data regarding QFR in the context of MI is sparse. In addition, QFR may be compromised in patients with MI due to diastolic and systolic dysfunction as well as microvascular dysfunction and neurohumoral activation, which may potentially alter local hemodynamics and reduce the reliability of QFR. Therefore, the aim of this study was to compare the diagnostic efficiency of QFR analysis based on acute angiograms (aQFR) with standard staged ischemia testing in the functional evaluation of non-culprit lesions of patients presenting with MI.

## Methods

### Patient selection

**Fig. 1:**
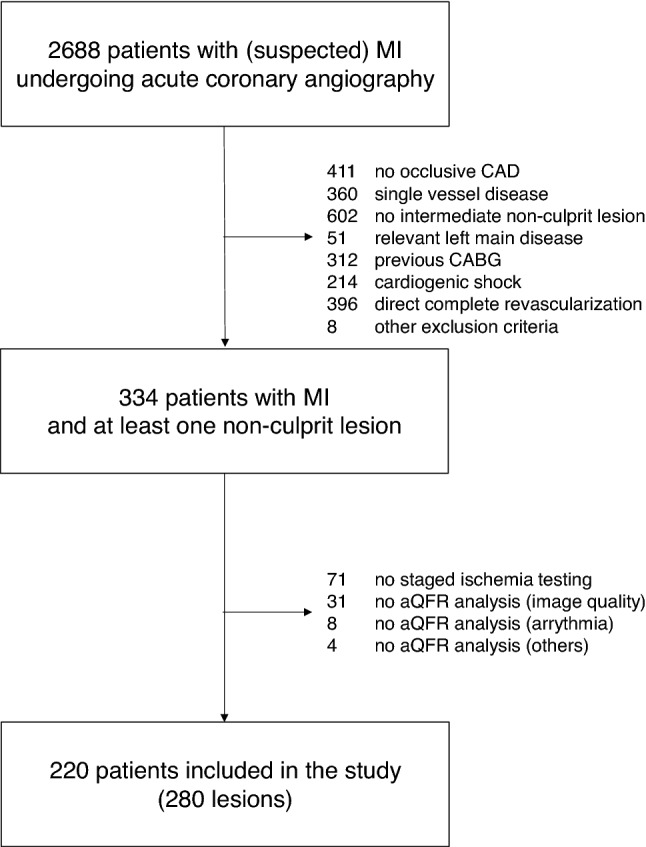
Details of study inclusion. Allowed ischemia tests were staged FFR, stress cardiac MRI or SPECT within 6 months of the initial angiography. Abbreviations: MI myocardial infarction; CAD coronary artery disease; CABG coronary artery bypass graft

We retrospectively analyzed 2688 patients who underwent an acute coronary angiography due to acute MI at the Cardiology Department of the University Hospital of the RWTH Aachen between the 1st January 2016 and the 1st June 2020. Inclusion criteria was the presence of one (or more) angiographically at least intermediate non-culprit lesion(s), which did not undergo PCI in the acute setting and were referred to planned ischemia testing at interventionalists discretion and based solely on visual assessment of the lesion; to be further included in the study, these lesions had an at least 40% diameter stenosis by retrospective quantitative angiography. Furthermore, non-culprit lesions had to be localized in a different vessel from the culprit lesion and to perfuse an area of viable myocardium. Exclusion criteria were absence of CAD, presence of 1-vessel CAD, absence of non-culprit lesions as defined above, relevant left main disease, previous CABG, cardiogenic shock during acute coronary angiography or direct indication for revascularization of the non-culprit vessel(s) by CABG or PCI without previous ischemia testing. Cardiogenic shock leading to study exclusion was defined as the need for vasopressors or assist devices to maintain a mean arterial pressure > 65 mmHg. Direct revascularization of non-culprit vessels was performed at the interventionalists discretion or following a Heart Team decision, e.g. due to visually severe (> 90%) stenosis and/or persistent angina with uncertain identification of the culprit lesion/vessel. Of the 334 resulting patients, 263 underwent planned myocardial ischemia testing to assess hemodynamic relevance of the remaining lesion(s) (Fig. 1). Ischemia testings included in the study protocol were (1) FFR measured in a staged coronary angiography, (2) stress cardiac MRI or (3) SPECT; these tests had to be performed within 6 months of the date of the acute MI. Of these 263 patients, 43 were excluded due to non-feasibility for aQFR analysis, which was in 31 cases due to insufficient image quality, in 8 cases due to arrhythmia and in 4 cases due to other reasons (among which chronic total occlusions and missing calibration). This resulted in 220 patients included in the study with 280 diseased vessels available for analysis. A flowchart depicting details of patient selection is shown in Fig. 1.

The study protocol was approved from the local Ethical Committee (EK 003-21) and is in accordance with the Declaration of Helsinki on ethical principles for medical research investigating human subjects.

### QFR analysis

**Fig. 2:**
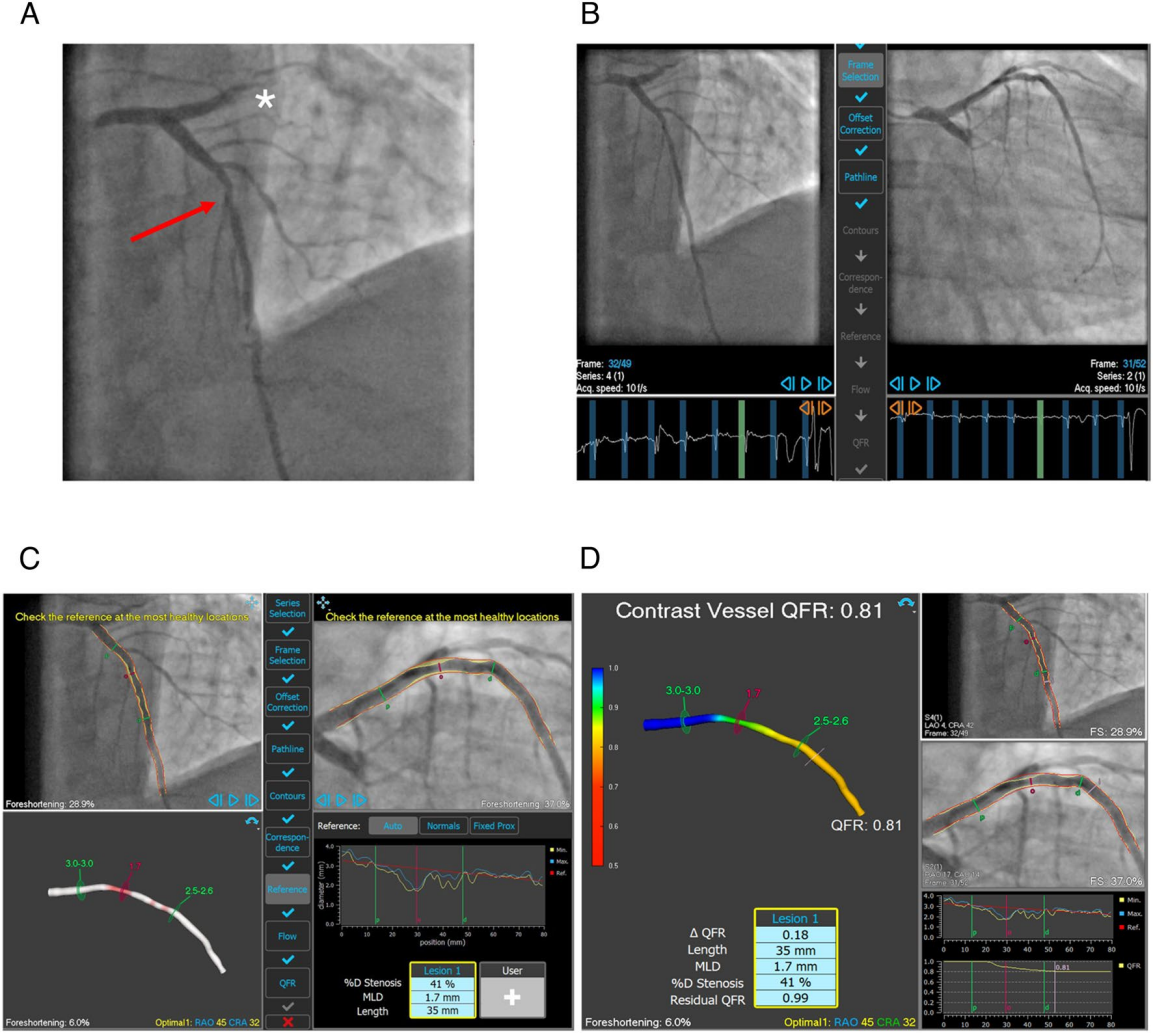
A representative QFR analysis of a non-culprit lesion in a patient with STEMI. In **A**, a cranial LAO projection shows total acute LCx occlusion (marked with a white asterisk) as well as an intermediate proximal LAD stenosis (indicated by the red arrow) as non-culprit lesion. In **B**, ECG-supported selection of end-diastolic frames from cranial LAO (right) and caudal RAO (right) views, both with good visualization of the non-culprit LAD stenosis. In **C**, automatic detection of vessel contours in the two projections (two upper quadrants), three-dimensional vessel reconstruction (right lower quadrant) and diameter profile (left lower quadrant). In **D**, the resulting QFR value (0.81) is shown, demonstrating no functional relevance of the non-culprit lesion.

QFR analysis was performed offline by a certified user (AM or RD) blinded to the results of ischemia testing. QFR analysis was performed using commercial software (QAngio XA 3D, Medis Medical Imaging System, Leiden, the Netherlands) employing a previously described protocol [13]. Two angiographic projections at least 25° apart with minimal overlap were used for QFR analysis. Due to the retrospective nature of the study, acquisition of coronary angiograms could not be performed with an optimized protocol for QFR analysis. Minimal acquisition rate required for inclusion was 10 frames/second. Quality of angiograms used for QFR analysis was defined qualitatively as “excellent” (no manual contour correction needed, very good contrast filling, no relevant foreshortening or vessel overlap), “good” (minimal manual contour correction needed, good contrast filling, minimal foreshortening or vessel overlap) or “acceptable” (> 3 points manual contour correction needed, acceptable contrast filling, some foreshortening or vessel overlap; all these factors did not impede performing QFR) at operator’s discretion. Frame-counting QFR was used for further analysis. An exemplificative analysis is shown in Fig. 2.

43 lesions (13.3% of the interrogated vessels) were not suitable for QFR analysis due to bad image quality or vessel overlap (31, 9.6% of the interrogated vessels), arrhythmia (8, 2.5%) or other causes (4, 1.2%). This resulted in 280 vessels from 220 patients with both aQFR analysis of the non-culprit lesion and planned myocardial ischemia testing (Fig. 1).

### Statistical analysis

Categorical variables were summarized as count (percentage), continuous variables as mean ± standard deviation. To identify the diagnostic efficiency of aQFR in predicting myocardial ischemia (as defined by staged ischemia testing), we performed receiver operating curve (ROC) analysis. A classification of the diagnostic efficacy according to the values of the area under the curve (AUC) was used as described elsewhere [17]. aQFR value with the highest Youden index (sensitivity + specificity− 1) was identified as optimal cut-off-value for predicting ischemia. Accuracy was defined as (true positives + true negative)/included lesions. Sensitivity and specificity, as derived from ROC analysis, were used to obtain cut-off values allowing a pre-specified sensitivity and specificity ≥ 95%. To compare the diagnostic efficacy of aQFR to other QCA-derived parameters as minimal luminal diameter (MLD) and percent area stenosis (%AS), we performed DeLong-tests, as previously described [18]. To assess association of aQFR with staged FFR values, we performed linear regression; data were also graphically expressed as Bland–Altman plot. All statistical analyses were performed with SPSS software v 26.0 (IBM Corp., Armonk, NY, USA). A *p* value < 0.05 was considered to indicate statistical significance.

## Results

### Patient and lesion characteristics

90 patients (40.9%) presented with STEMI at inclusion. Further patients characteristics are shown in Table 1.

**Table 1:** Patient characteristics

	*n* = 220
Age (yrs)	66.1 ± 14.0
Male sex (*n*, %)	165 (75)
STEMI at inclusion (*n*, %)	90 (40.9)
Culprit lesion localization	
LAD (*n*, %)	66 (30)
LCX (*n*, %)	49 (22.3)
RCA (*n*, %)	89 (40.4)
Diagonal branch (*n*, %)	3 (1.4)
Obtuse branch (*n*, %)	13 (5.9)
Extent of myocardial infarction	
Maximal TnT (pg/ml)	2993 ± 4317
Maximal CK (U/l)	1195 ± 1494
Maximal CK-MB (U/l)	151 ± 188
LVEF (%)	48.6 ± 8.5
Cardiovascular risk profile	
T2DM (*n*, %)	58 (26.4)
HbA1c (%)	6.2 ± 1.3
Hypertension (*n*, %)	143 (65.0)
Active nicotine use (*n*, %)	68 (30.9)
BMI (kg/m^2^)	27.1 ± 4.2
Cholesterol (mg/dl)	206 ± 124
LDLc (mg/dl)	137 ± 47
HDLc (mg/dl)	47 ± 14
Triglycerides (mg/dl)	110 ± 93

*TnT* high sensitive Troponin T, *T2DM* type 2 diabetes mellitus

Non-culprit vessels (*n* = 280) showed intermediate stenosis at quantitative coronary angiography (mean percentdiameter stenosis: 46.1 ± 9.0%). Hemodynamic relevance of lesions was assessed with FFR in 47 cases (16.8%), with cMRI in 200 cases (71.4%) and with SPECT in 33 cases (11.8%). These tests showed ischemia in 49 lesions (17.5%). Further lesion characteristics are reported in Table 2.

**Table 2:** Non-culprit lesion characteristics

	*n* = 280
Non-culprit lesion localization	
LAD (*n*, %)	106 (37.9)
LCx (*n*, %)	66 (23.6)
RCA (*n*, %)	64 (22.9)
Diagonal branch (*n*, %)	16 (5.7)
Obtuse branch (*n*, %)	23 (8.2)
Ramus Intermedius (*n*, %)	5 (1.8)
Lesion geometry	
MLD (mm)	1.37 ± 0.39
Percent diameter stenosis (%)	46.1 ± 9.0
Lesion length (mm)	23.8 ± 14.5
Functional assessment	
Staged assessment with FFR (*n*, %)	47 (16.8)
Staged assessment with cMRI (*n*, %)	200 (71.4)
Staged assessment with SPECT (*n*, %)	33 (11.8)
Presence of ischemia in staged test (*n*, %)	49 (17.5)
aQFR	0.85 ± 0.09
Quality of acute angiography	
Registration at 10 frames/s (*n*, %)	272 (97.1)
Registration at 15 frames/s (*n*, %)	8 (2.9)
Excellent imaging quality (*n*, %)	47 (16.8)
Good imaging quality (*n*, %)	206 (73.6)
Acceptable imaging quality (*n*, %)	27 (9.6)

*LAD* left descending artery, *LCx* circumflex artery, *RCA* right coronary artery, *MLD* minimal luminal diameter

### Diagnostic efficiency of aQFR in predicting ischemia

**Fig. 3:**
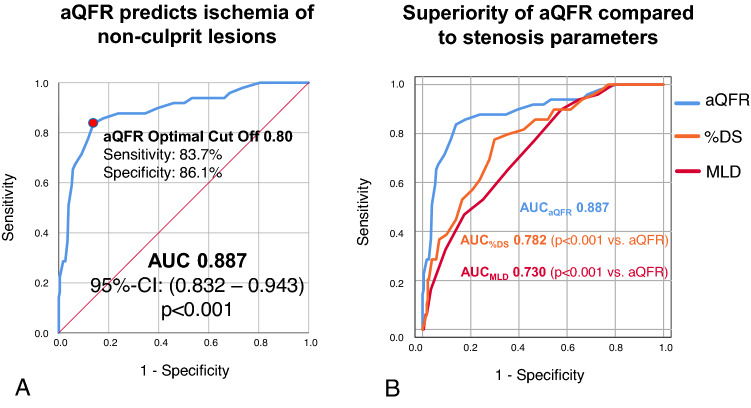
aQFR effectively predicts hemodynamic relevance of non-culprit lesions. In **A**, ROC curve shows excellent diagnostic efficiency of aQFR and the optimal aQFR cut-off-value for assessing ischemia of non-culprit lesions in patients with MI. In **B**, superiority of aQFR (blue line) compared to percent area stenosis (%DS, orange line) and minimal luminal diameter (MLD, red line) in the assessment of hemodynamic relevance of non-culprit lesions.

Accuracy of aQFR in the assessment of hemodynamic relevance of non-culprit lesions was 85.7%. aQFR could predict ischemia of non-culprit lesions with very good diagnostic efficiency (AUC = 0.887, 95% CI 0.832–0.943, *p* < 0.001). aQFR had an optimal cut-off for predicting ischemia of 0.80 (sensitivity = 83.7%, specificity = 86.1%). ROC curve for prediction of ischemia through aQFR is shown in Fig. 3A. Prediction of hemodynamic relevance of non-culprit lesion using aQFR was significantly superior to quantitative coronary angiography derived stenosis parameters such as minimal luminal diameter (AUC = 0.730, *p* < 0.001 vs. aQFR) and to percent area stenosis (AUC = 0.782, *p* < 0.001 vs. aQFR). Comparisons are shown in Fig. 3B.

### Head-to-head comparison of aQFR and staged FFR

**Fig. 4:**
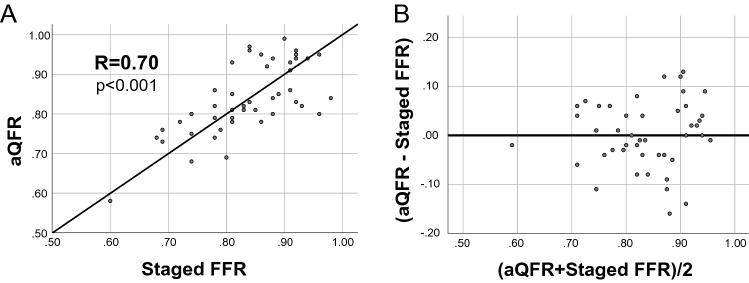
Head-to-head comparison of aQFR with staged FFR. In **A**, a strong linear correlation between aQFR and staged FFR of non-culprit lesions is shown. In **B**, Bland–Altman plot shows a good agreement of aQFR and staged FFR.

Based on the very good diagnostic efficiency of aQFR analysis in predicting ischemia of non-culprit lesions in patients with MI, we performed a head-to-head comparison of aQFR and staged FFR to further validate this method in lesions whose hemodynamic relevance was assessed with staged FFR (*n* = 46). We report no relevant difference between mean QFR (0.83 ± 0.09) and mean FFR (0.83 ± 0.08). QFR and FFR presented a strong positive linear correlation (*R* = 0.70, *p* < 0.001). Accuracy of aQFR in lesions analyzed with staged FFR was 83%. A graphical representation of this comparison is shown in Fig. 4.

### Development of a decision model combining aQFR and staged ischemia testing

**Fig. 5:**
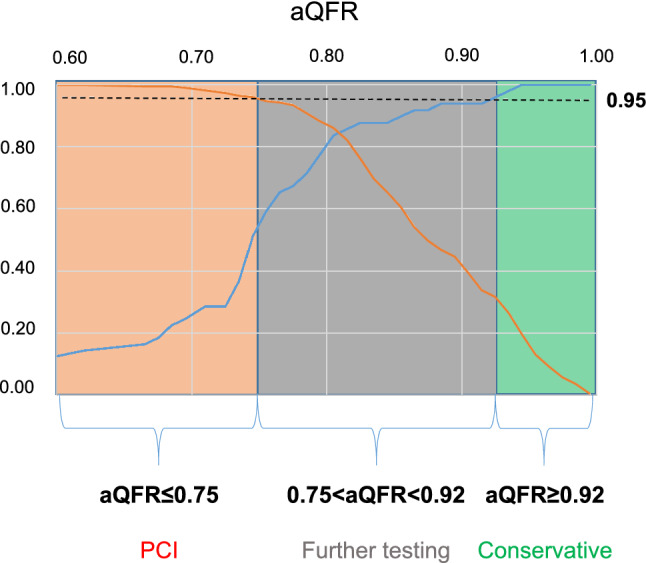
aQFR-based model for direct evaluation non-culprit lesions. Specificity (in red) and sensitivity (in blue) in predicting hemodynamic relevance of non-culprit lesions of patients with AMI are plotted in dependence of aQFR values; based on a (pre-specified) 95% sensitivity and 95% specificity level (which is marked by the black dashed line), we selected aQFR cut-offs. Therefore, we color-coded aQFR values in red (definite hemodynamic relevance), green (definite hemodynamic non-relevance) and grey (uncertain, further testing required)

After we saw a very good prediction of ischemia using aQFR, we aimed to develop a decision model based on aQFR yielding a 95% sensitivity and a 95% specificity in detecting ischemia. This could be obtained by defining a “grey zone” represented by lesions showing an aQFR between 0.75 and 0.92; these lesions need to be evaluated with further staged ischemia testing. A graphical representation of the model is shown in Fig. 5. This model could have allowed to avoid interrogating 122 vessels in 107 patients. By employing this decision algorithm, no staged ischemia testing would have been needed in 103 patients (46.8% of the total testing). In detail, our model would have avoided 13 staged angiographies with FFR (43.3%), 81 staged cMRI (51.3%) and 9 staged SPECT (33.3%) without a relevant loss in diagnostic efficacy.

## Discussion

The main finding of our study is the effectiveness of QFR analysis based on acute angiograms in assessing the functional relevance of intermediate non-culprit lesions in acute MI. In fact, aQFR showed very good accuracy and diagnostic efficiency to predict functional relevance of non-culprit lesions compared with standard staged ischemia testing (both invasive and non-invasive).

In patients with multivessel CAD and acute MI, current evidence favors treatment of all relevant coronary lesions, either during acute angiography or in staged procedures [2, 5–7, 9, 19]. However, assessment of hemodynamic relevance may require further tests, such as staged FFR measurement or imaging-based stress testing (as MRI or SPECT), especially in intermediate coronary lesions. A tempting strategy to minimize non-necessary testings could be the analysis of haemodynamic relevance of intermediate lesions based on acute angiograms by means of QFR.

### aQFR is feasible in acute MI

A possible objection to the use of aQFR may be due to the effects of local hemodynamics and microvascular dysfunction in the context of MI, which may lead to an underestimation of the relevance of non-culprit lesions. This parallels analogue objections on the use of FFR (or similar wire-based ischemia testing) in acute MI [20]. However, in our study, we could show in analogy to previous studies [21] a very good concordance of aQFR with staged FFR; in addition to our data Spitaleri et al. demonstrated a good association of aQFR with staged QFR [22] suggesting that these theoretical objections regarding the feasibility of aQFR are minor in the clinical setting.

Interestingly, the effectiveness of aQFR in assessing the hemodynamic relevance of non-culprit lesions was not hampered by the absence of an optimized protocol for the acquisition of angiograms and by the acquisition of angiograms at mostly 10 frames/second. Furthermore, in spite of the retrospective nature of our analysis, 16.8% of the interrogated vessel presented excellent image quality, and 90.4% an at least good quality. This implies that probably no time delay and no further contrast dye or radiation dose are necessary to enable aQFR, compared to the current standard of care in image acquisition during acute MI.

In the light of these data, it may be tempting to postulate that the changes in the hemodynamics occurring during acute MI seemingly do not significantly affect the diagnostic value of aQFR in the assessment of non-culprit lesions.

### aQFR effectively evaluates non-culprit lesions

Once shown that aQFR is not relevantly biased in the context of acute MI in our study, the diagnostic efficiency of this diagnostic tool in the assessment of non-culprit lesions has to be discussed. We could show that aQFR yields a very good diagnostic efficiency (AUC 0.89) compared to standard staged ischemia testing. The inclusion of both NSTEMI and STEMI patients as well as the presence of different modalities of ischemia testing (FFR, SPECT, cMRI) in comparison allow a good generalizability of our data. The finding confirms the important insights regarding the role of aQFR in assessment of non-culprit lesions offered by two previous pioneer studies [18, 23], which, however, only focused on STEMI patients and enrolled significantly smaller populations (respectively, 103 and 91 non-culprit lesions). Considering that NSTEMI is rapidly outgrowing STEMI as the most common presentation of MI [24], it is important to assess feasibility and effectiveness of aQFR throughout the whole spectrum of MI presentations. It has to be remarked that a prognostic effect of complete revascularization has been shown in randomized studies only for STEMI patients [2–5, 7–9]; however, data derived from large registers suggested a similar effect also in NSTEMI patients [6, 10–12], even though prospective evidence is scarce.

The very good diagnostic efficiency (AUC 0.89) of aQFR in this broad population may pave the way for a widespread use of this diagnostic tool in the assessment of non-culprit lesions in patients with MI. Interestingly, the AUC derived in our population for the prediction of ischemia of non-culprit lesions by aQFR is numerically similar to those reported in previous studies comparing QFR to FFR in patients with stable CAD (0.86 in the WIFI II trial [15]; 0.92 in the FAVOR II E-J trial [14]; 0.93 in the FAVOR China trial [16]). This suggests a similar efficacy of QFR independently of the clinical presentation of the patient (AMI vs. stable CAD).

### aQFR rationalizes staged ischemia testing for non-culprit lesions

The possibility to employ aQFR to limit staged ischemia testing without losing relevant sensitivity or specificity is a very tempting option. Staged ischemia testing is, in fact, time-consuming and cost-intensive [25]; furthermore, this is associated with possible complications, which is particularly true for invasive testing. Therefore, reserving staged ischemia testing for lesions which are not clearly classifiable by aQFR may allow a higher patient safety and comfort, as well as a better resource allocation. Our data show that a decision model based on aQFR may allow a first classification of non-culprit lesions: lesions with an aQFR ≤ 0.75 should be immediately referred for (staged) PCI, lesions with an aQFR ≥ 0.92 would need no further testing. Lesions within the “grey zone” (0.75–0.92) could benefit from additional testing to assess ischemia (see Fig. 5). This approach would have allowed, in our study, to reduce staged ischemia testing via CMR, SPECT or FFR by 46.8%. Although these specific cut-offs have to be evaluated in larger prospective studies, they show for the first time that an aQFR-based evaluation of non-culprit lesions may lead to a “rationalization” of ischemia testing, which in turn might limit complication and unnecessary procedures and consequently have a relevant structural and economic impact.

### Limitations

First of all, although the excellent accuracy of aQFR when compared to standard ischemia testing may allow to hypothesize similar prognostic effects of aQFR-guided revascularization strategies with the current standard of care, this needs to be evaluated in prospective studies. Due to the retrospective study design, patient enrollment was not based on a QCA assessment of the non-culprit lesion, but rather on visual assessment during acute angiography; still, in all patients included in our study QCA has been performed as part of the study protocol and showed at least intermediate stenosis.

Moreover, even though our aQFR-based decision model allows a “rationalization” of staged ischemia testing, the population directly benefiting from this strategy represents only a subset of the overall group of MI-patients, i.e. the patients with multivessel CAD and at least one intermediate stenosis not undergoing direct complete revascularization. This group represented, in our cohort, 12.4% of all patients with suspected MI and 14.6% of all patients with confirmed MI. Though, being able to optimize patient care and resource allocation in this small subgroup requiring higher care intensity is still a relevant need for clinicians, which may be at least partly met by employing aQFR for ruling out ischemia of non-culprit lesions.

Furthermore, due to possible effects of vasopressor or mechanical assist devices on coronary hemodynamics, we excluded patients with cardiogenic shock due to MI from our analysis. Therefore, we cannot draw any conclusion on this specific subpopulation. The same is true for patients with relevant left main disease, who were excluded due to study design.

## Conclusion

QFR analysis based on acute angiograms allows an effective assessment of functional relevance of non-culprit lesions in MI. This may avoid unnecessary diagnostic procedure and, as a consequence, avoid costs and patient risk. However, our findings need to be confirmed in larger, prospective studies.

## References

[1] Thygesen K (2018). Fourth universal definition of myocardial infarction (2018). Circulation.

[2] Escaned J (2018). Safety of the deferral of coronary revascularization on the basis of instantaneous wave-free ratio and fractional flow reserve measurements in stable coronary artery disease and acute coronary syndromes. JACC Cardiovasc Interv.

[3] Hakeem A (2016). Long-term prognosis of deferred acute coronary syndrome lesions based on nonischemic fractional flow reserve. J Am Coll Cardiol.

[4] Lee JM (2017). Prognosis of deferred non-culprit lesions according to fractional flow reserve in patients with acute coronary syndrome. EuroIntervention.

[5] Bainey KR (2014). Complete vs culprit-only revascularization for patients with multivessel disease undergoing primary percutaneous coronary intervention for ST-segment elevation myocardial infarction: a systematic review and meta-analysis. Am Heart J.

[6] Desperak P (2019). Long-term outcomes of patients with multivessel coronary artery disease presenting non-ST-segment elevation acute coronary syndromes. Cardiol J.

[7] Engstrom T (2015). Complete revascularisation versus treatment of the culprit lesion only in patients with ST-segment elevation myocardial infarction and multivessel disease (DANAMI-3-PRIMULTI): an open-label, randomised controlled trial. Lancet.

[8] Hassanin A (2015). Prognostic impact of multivessel versus culprit vessel only percutaneous intervention for patients with multivessel coronary artery disease presenting with acute coronary syndrome. EuroIntervention.

[9] Toma M (2010). Non-culprit coronary artery percutaneous coronary intervention during acute ST-segment elevation myocardial infarction: insights from the APEX-AMI trial. Eur Heart J.

[10] Bainey KR (2020). Long-term outcomes of complete revascularization with percutaneous coronary intervention in acute coronary syndromes. JACC Cardiovasc Interv.

[11] Kim MC (2020). Optimal revascularization strategy in non-st-segment-elevation myocardial infarction with multivessel coronary artery disease: culprit-only versus one-stage versus multistage revascularization. J Am Heart Assoc.

[12] Rathod KS (2018). Complete versus culprit-only lesion intervention in patients with acute coronary syndromes. J Am Coll Cardiol.

[13] Tu S (2016). Diagnostic accuracy of fast computational approaches to derive fractional flow reserve from diagnostic coronary angiography: the international multicenter FAVOR pilot study. JACC Cardiovasc Interv.

[14] Westra J (2018). Diagnostic performance of in-procedure angiography-derived quantitative flow reserve compared to pressure-derived fractional flow reserve: the FAVOR II Europe-Japan study. J Am Heart Assoc.

[15] Westra J (2018). Evaluation of coronary artery stenosis by quantitative flow ratio during invasive coronary angiography: the WIFI II study (wire-free functional imaging II). Circ Cardiovasc Imaging.

[16] Xu B (2017). Diagnostic accuracy of angiography-based quantitative flow ratio measurements for online assessment of coronary stenosis. J Am Coll Cardiol.

[17] Simundic AM (2009). Measures of diagnostic accuracy: basic definitions. EJIFCC.

[18] DeLong ER, DeLong DM, Clarke-Pearson DL (1988). Comparing the areas under two or more correlated receiver operating characteristic curves: a nonparametric approach. Biometrics.

[19] Smits PC (2020). Fractional flow reserve-guided multivessel angioplasty in myocardial infarction: three-year follow-up with cost benefit analysis of the compare-acute trial. EuroIntervention.

[20] Cuculi F (2014). Early change in invasive measures of microvascular function can predict myocardial recovery following PCI for ST-elevation myocardial infarction. Eur Heart J.

[21] Sejr-Hansen M (2019). Quantitative flow ratio for immediate assessment of nonculprit lesions in patients with ST-segment elevation myocardial infarction—an iSTEMI substudy. Catheter Cardiovasc Interv.

[22] Spitaleri G (2018). Quantitative flow ratio identifies nonculprit coronary lesions requiring revascularization in patients with ST-segment-elevation myocardial infarction and multivessel disease. Circ Cardiovasc Interv.

[23] Lauri FM (2020). Angiography-derived functional assessment of non-culprit coronary stenoses in primary percutaneous coronary intervention. EuroIntervention.

[24] Puymirat E (2017). Acute myocardial infarction: changes in patient characteristics, management, and 6 month outcomes over a period of 20 years in the FAST-MI program (French registry of acute ST-elevation or non-ST-elevation Myocardial Infarction) 1995 to 2015. Circulation.

[25] Kwong RY (2019). Cardiac magnetic resonance stress perfusion imaging for evaluation of patients with chest pain. J Am Coll Cardiol.

